# DATA in BRIEF of: Interventional Cardiac Catheterization in Neonatal Age: Results in a Multi-centre Italian Experience

**DOI:** 10.1016/j.dib.2020.105694

**Published:** 2020-05-13

**Authors:** Mario Giordano, Giuseppe Santoro, Gabriella Agnoletti, Mario Carminati, Andrea Donti, Paolo Guccione, Maurizio Marasini, Ornella Milanesi, Biagio Castaldi, Martino Cheli, Roberto Formigari, Gianpiero Gaio, Luca Giugno, Alessia Lunardini, Carlotta Pepino, Maria Giovanna Russo, Isabella Spadoni

**Affiliations:** a Pediatric Cardiology, “Ospedali dei Colli”, University of Campania “Luigi Vanvitelli”, Naples; b Pediatric Cardiology and GUCH Unit, “Ospedale del Cuore”, Tuscany-CNR Foundation “G. Monasterio”, Massa; c Pediatric Cardiology, “Regina Margherita” Hospital, University of Turin; d Pediatric Cardiology and GUCH Unit, IRCCS Policlinico San Donato, Milan; e Pediatric Cardiology and GUCH Unit, “S. Orsola-Malpighi” Hospital, University of Bologna; f Pediatric Cardiology, “Bambino Gesù” Hospital, Rome; g Pediatric Cardiology, IRCCS “G. Gaslini” Hospital, Genoa; h Pediatric Cardiology, University of Padua

**Keywords:** Interventional Cardiac Catheterization, Neonate, Adverse Events, Mortality, Risk Factor, Multivariable Analysis

## Abstract

A comprehensive description of morbidity and mortality as well as risk factors of interventional cardiac catheterization performed in neonatal age was reported in our paper recently published on the **International Journal of Cardiology** (**IJCA28502; PII: S0167-5273(20)30384-3; DOI: 10.1016/j.ijcard.2020.04.013**). Eight Italian high-volume centres of Paediatric Cardiology were involved in this observational, retrospective data collection and analysis. In this dataset, clinical and procedural characteristics of 1423 newborns submitted to 1551 interventional cardiac catheterization procedures were analyzed. Primary outcomes were considered procedure and in-hospital mortality as well as major adverse event and procedural failure rates. Secondary outcomes were considered minor adverse events and need for blood transfusion. Targets of this data analysis were: 1) to evaluate the overall major risk factors of interventional cardiac catheterization; 2) to identify the most hazardous interventional procedures; 3) to assess possible trends of individual procedures as well as their outcome over time; 4) to find possible relationships between the volume activity of any centre and the procedure and follow-up outcome. In particular, this Data in Brief companion paper aims to report the specific statistic highlights of the multivariable analysis (binary logistic regression) used to assess the impact of any potential risk factors on the type of procedure over a short-term follow-up.

Specifications table

**Table:** 

Subject	Cardiology and Cardiovascular Medicine
Specific subject area	Interventional Cardiology, Congenital Heart Disease, Neonatology, Morbidity and Mortality
Type of data	Table, Figure
How data were acquired	Clinicians’ analysis recording single centre registries
Data format	RAW
Parameters for data collection	Sample: Interventional cardiac catheterizations in neonatal age Parameters: centre, sex gender, weight, age, prematurity, co-morbidity, genetic syndrome, congenital heart disease, interventional procedure, hybrid approach, procedure failure, adverse events, mortality, blood transfusion
Description of data collection	Retrospective collection by analysing the procedural registry of each centre. No experimental features were used or applied to data collection and analysis.
Data source location	Bologna, Genoa, Massa, Milan, Naples, Padua, Rome, Turin (Italy)
Data accessibility	In the ARTICLE as well as in the SUPPLEMENTARY FILE section
Related research article	**Interventional Cardiac Catheterization in Neonatal Age: Results in a Multicentre Italian Experience** Giordano M, Santoro G, Agnoletti G, Carminati M, Donti A, Guccione P, Marasini M, Milanesi O, Castaldi B, Cheli M, Formigari R, Gaio G, Giugno L, Lunardini A, Pepino C, Russo MG, Spadoni I *Int J Cardiol* 2020; PII: S0167-5273(20)30384-3; DOI: 10.1016/j.ijcard.2020.04.013 (In press)

## Value of the data

• Interventional cardiac catheterization is an increasing approach to treat newborns with critical congenital heart disease. No data about risk stratification of interventional procedures in this subset of patients are so far reported in literature. Our dataset aims to evaluate the intrinsic risk of trans-catheter interventional approach as well as the potential risk factors involved in any individual procedure performed at this age.
• The nationwide cohort dataset recently published in the related research article provides specific information on morbidity and mortality of newborns submitted to interventional cardiac catheterization. The Authors showed that the morbidity (major adverse events and procedural failure) is significantly related to the complexity of the intended procedure while the in-hospital mortality significantly depends on the clinical characteristics and hemodynamic stability of the patient. These data may be useful to cardiologists involved in the management of newborns affected by congenital heart disease to clearly understand patient's risk profile of any interventional procedure.
• The safety and effectiveness data of trans-catheter approach reported in this Data in Brief paper and its related research article may hopefully promote further developments in trans-catheter treatment of neonates with critical congenital heart disease. “Ad hoc”-planned future researches aiming to specifically compare percutaneous and surgical approaches in this subset of patients will give further useful information to set the future guide-lines of management of critical, neonatal-onset cardiac malformations.
• Defining careful risk profile of newborns in whom an interventional cardiac catheterization is planned allows to improve pre-procedure counselling with parents and care-givers as well as gives further insights about the short-term prognosis of these frail patients. These data will hopefully improve timing and type of interventional approach (percutaneous vs surgical vs hybrid) in this frail subset of patients.

## 1 Data Description

This dataset (see also the SUPPPLEMENTARY FILE section) gives relevant details and explanations about the enrolled population/procedures (catheterizations/procedures and adverse events) and statistical analysis techniques (mainly multi-variable analysis). These data are expressed as figures and tables as well as in form of RAW DATA in the SUPPPLEMENTARY FILE section:

- the Table 1 describes the different catheterization sessions and interventional procedures performed in our cohort

**Table 1:** Summary catheterizations and procedures

Interventional catheterization	N (%)	Interventional procedure	N (%)
**Total catheterizations**	**1551,00**	**Total procedures**	**1615,00**
Rashkind	665 (42.9)	Rashkind	692 (42.8)
BPV	335 (21.6)	BPV	354 (21.9)
AD stent	169 (10.9)	AD stent	211 (13.1)
BAV	130 (8.4)	BAV	135 (18.4)
APV Perforation	114 (7.4)	APV Perforation	126 (18.2)
RVOT stent	16 (1.0)	RVOT stent	16 (1.0)
IVC/SVC PTA	10 (0.6)	IVC/SVC PTA	11 (0.7)
MAPCAs embolization	7 (0.5)	IAS Perforation	9 (0.6)
RPA/LPA PTA	6 (0.4)	IAS stent	9 (0.6)
Surgical Shunt stent	6 (0.4)	MAPCAs embolization	8 (0.5)
Aorta PTA	5 (0.3)	RPA/LPA PTA	8 (0.5)
AD embolization	5 (0.3)	Aorta PTA	7 (0.4)
IAS Perforation	5 (0.3)	Surgical Shunt stent	6 (0.4)
RPA/LPA stent	5 (0.3)	AD embolization	6 (0.4)
Thrombolysis	3 (0.2)	RPA/LPA stent	6 (0.4)
IAS stent	2 (0.1)	Thrombolysis	3 (0.2)
Surgical Shunt PTA	2 (0.1)	Surgical Shunt PTA	2 (0.1)
AD stent PTA	2 (0.1)	AD stent PTA	2 (0.1)
PV PTA	1 (<0.1)	PV PTA	1 (<0.1)
Aorta stent	1 (<0.1)	Aorta stent	1 (<0.1)
Femoral artery stent	1 (<0.1)	Femoral artery stent	1 (<0.1)
AD stent + Rashkind	14 (0.9)	BTV	1 (<0.1)
BPV + AD stent	12 (0.7)		
APV perf + AD stent	7 (0.5)		
APV perf + Rashkind	4 (0.3)		
IAS Perforation + IAS stent	4 (0.3)		
BPV + Rashkind	3 (0.2)		
AD stent + RPA/LAP stent	2 (0.1)		
BAV + Rashkind	2 (0.1)		
BAV + AD stent	2 (0.1)		
Rashkind + IAS stent	2 (0.1)		
BAV + BPV	1 (<0.1)		
AD stent + Aorta PTA	1 (<0.1)		
APV perf + RPA/LAP PTA	1 (<0.1)		
MAPCAs embolization + AD embolization	1 (<0.1)		
BPV + AD stent + IVC PTA	1 (<0.1)		
IAS stent + AD stent	1 (<0.1)		
BPV + AD stent + Rashkind	1 (<0.1)		
Rashkind + Aorta PTA	1 (<0.1)		
BPV + BTV + AD stent	1 (<0.1)		

**AD**: Arterial Duct; **APV**: Atretic Pulmonary Valve; **BAV**: Balloon Aortic Valvuloplasty; **BPV**: Balloon Pulmonary Valvuloplasty; **BTV**: Balloon Tricuspid Valvuloplasty; **IAS**: InterAtrial Septum; **IVC**: Inferior Vena Cava; **LPA**: Left Pulmonary Artery; **MAPCA**: Major Aorto-Pulmonary Collateral Arteries; **PTA**: Percutaneous Trans-luminal Angioplasty; **PV**: Pulmonary Vein; **RPA**: Right Pulmonary Artery; **RVOT**: Right Ventricle Outflow Tract; **SVC**: Superior Vena Cava
- the Table 2 labels the adverse events (either major or minor) listed in 8 categories: vascular access adverse events, arrhythmias, pericardial effusions, direct intra-cardiac lesions, great vessels damages, technical complications of the procedure, significant hemodynamic compromise and other adverse events

**Table 2:** Summary Adverse Events

Major and Minor Adverse Events	N (%)
**Vascular Access Adverse Events**	**21 (13.3)**
Femoral Artery Pseudo-aneurysm	3 (1.9)
Femoral Artery Thrombosis	12 (7.6)
Femoral Vein Thrombosis	6 (3.8)
**Arrhythmias**	**23 (14.6)**
Brady-arrhythmia	7 (4.4)
Atrial Flutter	7 (4.4)
Supra-ventricular Tachycardia	5 (3.2)
Ventricular Fibrillation	4 (2.5)
**Pericardial Effusion**	**38 (24.1)**
Haematic Pericardial Effusion	26 (16.5)
Cardiac Tamponade	12 (7.6)
**Direct Intracardiac Lesions**	**8 (5.1)**
Ventricular Pseudo-aneurysm	2 (1.3)
Heart Perforation	3 (1.9)
Rope Rupture with severe TR	1 (0.6)
Intra-cardiac Thrombus	2 (1.3)
**Great Vessels Damage**	**9 (5.6)**
Aortic Dissection	1 (0.6)
RPA/LPA Stenosis	3 (1.9)
IVC/SVC Perforation	2 (1.3)
RPA Perforation	1 (0.6)
Acute SVC Thrombosis	1 (0.6)
SVC Thrombosis	1 (0.6)
**Technical Complications of Procedure**	**9 (5.6)**
Stent Embolization	4 (2.5)
Balloon Embolization	1 (0.6)
Stent Jailing	1 (0.6)
Acute Intra-stent Thrombosis	3 (1.9)
**Significant Hemodynamic Compromise**	**26 (16.5)**
Cardio-circulatory Arrest	4 (2.5)
Low-output Syndrome	14 (8.9)
Shock	8 (5.1)
**Others**	**24 (15.2)**
Pulmonary Embolism	1 (0.6)
Mild Haemorrhage	2 (1.3)
Transient Myocardial Ischemia	6 (3.8)
Cerebral Ischemia	4 (2.5)
Pneumothorax	5 (3.2)
Sepsis	5 (3.2)
Pleural Effusion	1 (0.6)
**Total Adverse Events**	**158,00**

**IVC**: Inferior Vena Cava; **LPA**: Left Pulmonary Artery; **RPA**: Right Pulmonary Artery; **SVC**: Superior Vena Cava; **TR**: Tricuspid Regurgitation
- the Table 3 and the Table 4 show multi-variable analyses (binary logistic regression) of the potential risk factors (gender, low-weight, prematurity, genetic syndrome, uni-ventricular heart physiology, hybrid approach, risk category, age ≤7 days and procedure failure) and the major interventional procedures (arterial duct stenting, atretic pulmonary valve perforation, balloon aortic valvuloplasty, balloon pulmonary valvuloplasty, Rashkind atrioseptostomy) in terms of primary and secondary outcomes

**Table 3:** Multi-variable analysis of the potential risk factors

Binary logistic regression of the primary outcomes												
	FAILURE			MAE			MORTALITY			COMPOSITE OUTCOME		
	Wald	OR (95% CI)	p-	Wald	OR (95% CI)	p-	Wald	OR (95% CI)	p-	Wald	OR (95% CI)	p-
**Gender**	0.80	1.28 (0.75 – 2.21)	0.37	1.04	0.77 (0.46 – 1.28)	0.31	3.67	0.58 (0.33 – 1.01)	0.06	0.87	0.84 (0.58 – 1.21)	0.08
**LW (≤2.5 kg)**	1.20	1.48 (0.74 – 2.96)	0.27	5.05	1.99 (1.09 – 3.61)	0.03	10.67	2.75 (1.50 – 5.04)	**<0.01**	9.11	1.96 (1.27 – 3.04)	**<0.01**
**Prematurity**	0.06	0.88 (0.45 – 3.35)	0.81	0.23	1.22 (0.55 – 2.71)	0.63	9.41	3.09 (1.50 – 6.34)	**<0.01**	6.15	2.02 (1.16 – 3.52)	**<0.01**
**Genetic Syndromes**	0.71	0.42 (0.54 – 3.21)	0.42	4.01	2.73 (1.02 – 7.27)	0.05	20.86	7.88 (3.25 – 19.12)	**<0.01**	6.51	2.73 (1.26 – 5.90)	**<0.01**
**UVH**	16.19	3.81 (1.99 – 7.30)	**<0.01**	0.55	1.30 (0.65 – 2.60)	0.46	31.59	5.35 (2.98 – 9.60)	**<0.01**	32.94	3.78 (2.40 – 5.96)	**<0.01**
**Hybrid Approach**	2.69	0.27 (0.06 – 3.21)	0.10	1.48	1.88 (0.68 – 5.2)	0.22	0.03	1.10 (0.38 – 3.17)	0.86	0.99	0.65 (0.28 – 1.51)	0.91
**Risk Category**	28.49	4.67 (2.65 – 8.23)	**<0.01**	14.94	2.80 (1.66 – 4.72)	**<0.01**	0.03	1.06 (0.60 – 1.85)	0.86	39.22	3.22 (2.23 – 4.64)	**<0.01**
**Age ≤7 days**	6.70	2.36 (1.23 – 4.54)	**<0.01**	1.39	1.39 (0.81 – 2.39)	0.24	0.11	0.91 (0.53 – 1.58)	0.74	6.92	1.70 (1.14 – 2.53)	**<0.01**
**Failure**	-	-	-	36.75	7.79 (4.01 – 15.12)	**<0.01**	49.53	13.20 (6.43 – 27.07)	**<0.01**	-	-	-

Binary logistic regression of the secondary outcomes												
	BLOOD TRANSFUSION			MiAE								
	Wald	OR (95% CI)	p-	Wald	OR (95% CI)	p-						
**Gender**	3.51	0.59 (0.33 – 1.06)	0.08	0.79	0.80 (0.50 – 1.30)	0.37						
**LW (≤2.5 kg)**	6.40	2.28 (1.20 – 4.30)	**<0.01**	0.21	0.85 (0.43 – 1.70)	0.65						
**Prematurity**	3.26	2.01 (0.94 – 4.30)	0.07	0.06	0.88 (0.32 – 2.43)	0.80						
**Genetic Syndromes**	10.00	4.12 (1.71 – 9.93)	**<0.01**	0.01	1.04 (0.24 – 4.52)	0.96						
**UVH**	0.01	0.98 (0.43 – 2.21)	0.95	1.14	0.59 (0.23 – 1.55)	0.29						
**Hybrid Approach**	0.04	0.89 (0.26 – 3.03)	0.85	0.51	0.47 (0.06 – 3.77)	0.48						
**Risk Category**	17.76	3.47 (1.94 – 6.18)	**<0.01**	3.32	1.57 (0.97 – 2.56)	0.07						
**Age ≤7 days**	7.58	0.46 (0.27 – 0.80)	**<0.01**	0.13	1.10 (0.66 – 1.83)	0.72						
**Failure**	5.36	2.90 (1.18 – 7.16)	0.02	1.02	1.66 (0.62 – 4.40)	0.31						

**Abbreviations. LW**: Low-Weight; **MAE**: Major Adverse Events; **MiAE**: Minor Adverse Events; **UVH**: Uni-Ventricular Heart

**Table 4:** Multi-variable analysis of the major procedures

Binary logistic regression of the primary outcomes												
	FAILURE			MAE			MORTALITY			COMPOSITE OUTCOME		
	Wald	OR (95% CI)	p-	Wald	OR (95% CI)	p-	Wald	OR (95% CI)	p-	Wald	OR (95% CI)	p-
**AD Stenting**	0.16	0.74 (0.17 – 3.18)	0.69	7.71	3.87 (1.49 – 10.07)	**<0.01**	3.76	2.99 (0.99 – 9.02)	0.05	8.70	3.13 (1.47 – 6.66)	**<0.01**
**APV Perforation**	14.49	17.92 (4.05 – 79.16)	**<0.01**	9.63	5.56 (1.88 – 16.43)	**<0.01**	0.59	1.67 (0.45 – 6.17)	0.44	20.51	7.21 (3.07 – 16.95)	**<0.01**
**BAV**	1.68	3.10 (0.56 – 17.12)	0.2	8.73	5.71 (1.80 – 18.15)	**<0.01**	2.59	2.93 (0.79 – 10.80)	0.11	11.42	4.84 (1.94 – 12.09)	**<0.01**
**BPV**	0.01	0.93 (0.16 – 5.35)	0.93	0.73	0.56 (0.15 – 2.12)	0.39	3.41	0.19 (0.03 – 1.10)	0.06	1.03	0.60 (0.23 – 1.61)	0.31
**Rashkind Atrio-septostomy**	1.02	2.15 (0.48 – 9.58)	0.31	1.70	2.01 (0.7 – 5.74)	0.19	1.31	1.99 (0.61 – 6.49)	0.25	4.64	2.46 (1.08 – 5.60)	0.03

Binary logistic regression of the secondary outcomes												
	BLOOD TRANSFUSION			MiAE								
	Wald	OR (95% CI)	p-	Wald	OR (95% CI)	p-						
**AD Stenting**	5.93	4.10 (1.31 – 12.25)	0.02	0.44	1.44 (0.49 – 4.18)	0.51						
**APV Perforation**	0.27	1.42 (0.38 – 5.30)	0.6	3.38	3.06 (0.93 – 10.10)	0.07						
**BAV**	2.85	3.05 (0.84 – 11.09)	0.09	1.75	0.30 (0.05 – 1.79)	0.19						
**BPV**	0.00	1.01 (0.30 – 3.38)	1.0	0.35	1.43 (0.44 – 4.66)	0.55						
**Rashkind Atrio-septostomy**	3.10	0.31 (0.08 – 1.15)	0.08	0.35	0.70 (0.21 – 2.92)	0.56						

**Abbreviations. AD**: Arterial Duct; **APV**: Atretic Pulmonary Valve; **BAV**: Balloon Aortic Valvuloplasty; **BPV**: Balloon Pulmonary Valvuloplasty; **MAE**: Major Adverse Events; **MiAE**: Minor Adverse Events
- the Table 5 describes in each large column the multi-variable analysis (binary logistic regression) of the different potential risk factors in terms of composite outcome (in-hospital mortality, major adverse event and/or failure) of each major procedure, as individually analyzed

**Table 5:** Multi-variable analysis of the potential risk factors in the most common procedures and hybrid approaches

Binary logistic regression of the composite outcome (failure and/or major adverse events and/or mortality)												
	AD Stenting			Atretic Pulmonary Valve Perforation			Rashkind Atrio-septostomy			Balloon Pulmonary Valvuloplasty		
	Wald	OR (95% CI)	p-	Wald	OR (95% CI)	p-	Wald	OR (95% CI)	p-	Wald	OR (95% CI)	p-
**Gender**	0.40	1.35 (0.54 – 3.40)	0.53	2.93	0.45 (0.18 – 1.12)	0.09	0.36	0.83 (0.45 – 1.54)	0.55	0.90	0.50 (0.12 – 2.10)	0.34
**LW (≤2.5 kg)**	1.39	1.95 (0.64 – 5.92)	0.24	5.46	3.46 (1.22 – 9.80)	0.02	1.35	1.56 (0.74 – 3.33)	0.25	0.93	2.27 (0.43 – 12.06)	0.34
**Prematurity**	0.14	1.32 (0.30 – 5.85)	0.71	0.08	1.22 (0.30 – 4.98)	0.78	6.08	3.23 (1.27 – 8.22)	0.02	1.62	3.50 (0.51 – 24.08)	0.20
**Genetic Syndromes**	7.72	5.24 (1.63 – 16.83)	**<0.01**	-	-	-	4.36	4.54 (1.10 – 18.82)	0.04	-	-	-
**UVH**	7.86	3.79 (1.49 – 9.63)	**<0.01**	-	-	-	21.66	4.71 (2.45 – 9.05)	**<0.01**	-	-	-
**Age ≤7 days**	0.76	1.52 (0.59 – 3.92)	0.38	0.03	0.89 (0.25 – 3.24)	0.86	1.11	1.77 (0.61 – 5.14)	0.29	<0.01	1.03 (0.27 – 3.97)	0.97

	Balloon Aortic Valvuloplasty			Hybrid Approach								
	Wald	OR (95% CI)	p-	Wald	OR (95% CI)	p-						
**Gender**	2.97	2.58 (0.88 – 7.58)	0.09	0.19	0.62 (0.07 – 5.25)	0.66						
**LW (≤2.5 kg)**	7.59	6.30 (1.70 – 23.34)	**<0.01**	0.02	1.15 (0.15 – 8.86)	0.90						
**Prematurity**	0.29	0.66 (0.15 – 3.01)	0.59	0.07	1.44 (0.10 – 21.88)	0.79						
**Genetic Syndromes**	-	-	-	2.53	10.23 (0.58 – 179.9)	0.11						
**UVH**	0.20	1.46 (0.27 – 7.75)	0.66	2.51	0.27 (0.05 – 1.37)	0.11						
**Age ≤7 days**	4.86	4.21 (1.17 – 15.12)	0.03	2.89	4.16 (0.80 – 21.52)	0.09						

**Abbreviations. LW**: Low-Weight; **UVH**: Uni-Ventricular Heart
- the Table 6 compares the first and second half-time periods (2000-2008 vs 2009-2017) of our observational dataset in terms of demography, risk factors and interventional procedures

**Table 6:** Comparison of temporal period (years 2000-2008 vs 2009-2017)

	Years 2000-2008	Years 2009-2017	p-value
Total catheterization	N=528	N=1023	
**Risk Factors and Demographic Data**			
**Weight (kg)**	3.0±0.5	3.0±0.6	0.8
**Prematurity**	27 (5.1%)	89 (8.7%)	**<0.01**
**Genetic syndromes**	6 (1.1%)	34 (3.3%)	**<0.01**
**UVH physiology**	40 (7.8%)	113 (11%)	0.03
**Hybrid Approach**	2 (0.4%)	40 (3.9%)	**<0.01**
**Outcomes Analysis**			
**Composite Outcomes**	46 (8.7%)	114 (11.1%)	0.1
**Failure**	19 (4.2%)	40 (3.9%)	0.8
**MAE**	22 (4.2%)	55 (5.4%)	0.3
**Mortality**	20 (3.8%)	60 (5.9%)	0.08
**MiAE**	23 (4.4%)	58 (5.7%)	0.3
**Blood transfusion**	13 (2.5%)	51 (5.0%)	0.02
**Total procedures**	**N=537**	**N=1078**	
**AD stenting**	9 (1.7%)	173 (16.0%)	**<0.01**
**APV perforation**	49 (9.1%)	77 (7.1%)	0.2
**BAV**	51 (9.5%)	84 (7.8%)	0.2
**BPV**	116 (21.6%)	238 (22.0%)	0.8
**Rashkind Atrio-septostomy**	266 (49.5%)	426 (39.5%)	**<0.01**
**RVOT stenting**	2 (0.4%)	14 (1.3%)	0.08

Continuous variables are expressed as mean±SD, whereas dichotomic variables as absolute values (percentage). Test T-Student and chi-square test were used to compare continuous and dichotomic variables, respectively.

**Fig. 1:**
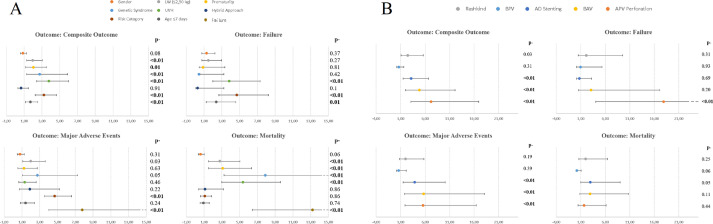
Forest plots reporting the effects of potential risk factors (**A**) and major procedures (**B**) on the primary outcomes.
- the Figure 2 shows, anonymously, the number of trans-catheter interventions for single centre (**A**) and, accordingly, the rate of composite outcome (**B**)

**Abbreviations. AD**: Arterial Duct; **APV**: Atretic Pulmonary Valve; **BAV**: Balloon Aortic Valvuloplasty; **BPV**: Balloon Pulmonary Valvuloplasty; **MAE**: Major Adverse Events; **MiAE**: Minor Adverse Events; **RVOT**: Right Ventricle Outflow Tract; **UVH**: UniVentricular Heart
- the Figure 1 is the forest plots representation of multi-variable analysis of the potential risk factors (**A**) and the most performed procedures (**B**) on the primary outcomes

**Fig. 2:**
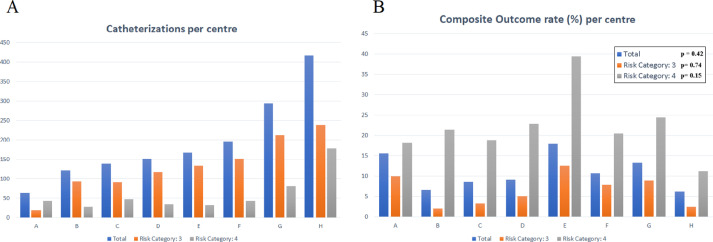
Column graph of the number of interventional catheterizations (**A**) and the composite outcome rate (**B**) for any individual centre both as overall (**blue column**) and separated data ranked as lower (**orange column**) and higher (**grey column**) procedure risk. The box reported the p-value calculated by linear regression analysis test.

## 2 Experimental Design, Materials, and Methods

In the related research article [1], a retrospective detection of all consecutive interventional cardiac catheterizations performed in neonatal age was carried out by the eight Italian higher-volume centres involved in the study (Bologna, Genoa, Massa, Milan, Naples, Padua, Rome and Turin). To achieve this dataset, hospital registry and clinical folders were examined. From January 2000 to December 2017, 1423 consecutive newborns were submitted to 1551 interventional cardiac catheterizations, during which 1615 interventions were performed. The term “catheterization” was used to indicate any procedural session, while the term “procedure” was used to report any specific intervention. Primary outcomes were any procedure-related major adverse event (MAE), in-hospital mortality and failure of the intended procedure. They were analyzed both individually and as a composite outcome. Secondary outcomes were any procedure-related minor adverse event (MiAE) and need for blood transfusion. Gender, low-weight, prematurity, genetic syndrome, uni-ventricular heart physiology, hybrid approach, risk category, age ≤7 days and failure were analyzed as potential risk factors.

Multi-variable analysis was performed with a binary logistic regression [2] and used to evaluate the independent impact of any risk factor on the outcome of interventional cardiac catheterization, either as a whole or for each specific procedure. Furthermore, the multi-variable analysis was used to evaluate the risk profile of the five more common procedures (arterial duct stenting, atretic pulmonary valve perforation, balloon aortic valvuloplasty, balloon pulmonary valvuloplasty, Rashkind atrio-septostomy) on short-term outcome.

The data reported in the Table 6, comparing the first and the second half observational period were analysed by two-tail chi-square test (for categorical and binary variables) or unpaired two-samples Student's t-test (for continuous variables).

The data were then divided for any centre in order to evaluate, by linear regression test, the impact of the volume of activity of any individual centre on the composite outcome. The same statistical analysis was also made by separating the higher-risk procedures (risk category 4) from the lower ones (risk category 3) [3].

## Appendix Supplementary materials

Image, application 1

Image, application 2
